# Demographic characteristics and short-term outcomes of laparoscopic colon cancer surgeries at a newly developed cancer center in Peshawar, Pakistan

**DOI:** 10.12669/pjms.40.5.8732

**Published:** 2024

**Authors:** Riaz Ahmad, Hussain Jan Abbasi, Irfan Ul Islam Nasir, Muhammad Fahd Shah

**Affiliations:** 1 Riaz Ahmad Fellow Colorectal Surgery, Shaukat Khanum Memorial Cancer Institute and Research Centre, Peshawar, Pakistan; 2 Hussain Jan Abbasi Fellow Colorectal Surgery, Shaukat Khanum Memorial Cancer Institute and Research Centre, Peshawar, Pakistan; 3 Irfan Ul Islam Nasir Consultant Surgical Oncologist, Shaukat Khanum Memorial Cancer Institute and Research Centre, Peshawar, Pakistan; 4 Muhammad Fahd Shah Consultant Surgical Oncologist, Shaukat Khanum Memorial Cancer Institute and Research Centre, Peshawar, Pakistan

**Keywords:** Colorectal cancer, CRC, Incidence, Mortality with CRC in Pakistan

## Abstract

**Objective:**

In Pakistan, colon cancer ranks fourth in incidence, exhibiting survival rates of 90% to 14%, contingent on TNM staging and early detection. This research focuses on the demographic involvement and short-term outcomes of elective colon cancer resections at a newly established tertiary care cancer center utilizing laparoscopic procedures.

**Method:**

A retrospective analysis of elective laparoscopic colorectal resections at Shaukat Khanum Memorial Cancer Institute and Research Centre, Peshawar, from April 2021 to February 2022 was conducted. Out of 157 cases, 79 had colon cancer. Criteria included patients >18 years old with positive biopsies; consent non-providers were excluded. Statistical analysis employed descriptive statistics and cross-tabulations using SPSS-22.

**Results:**

The study encompassed biopsy-confirmed colon cancers in patients >18 years. 157 colorectal cases were performed, including 79 colon cancers. The sample comprised 61 males (77.2%) and 18 females (22.7%), mean age 42 years. Most patients (33%) were in the 36-45 age group. Majority were from KPK (69.6%), followed by Afghanistan (24%). Tumors were predominantly in the ascending colon (30.3%). Most were moderately differentiated (70.8%). Mean lymph node yield was 19.0, with 1.2% requiring open laparotomy. Post-discharge, one readmission occurred within 30 days. Mortality within 90 days was 2%, attributed to aspiration pneumonia and chemotherapy-related effects.

**Conclusions:**

Elective laparoscopic colonic surgery exhibits safety and efficacy in treating colon cancer. The study provides evidence of minimal morbidity and mortality, low readmission rates, and absence of anastomotic leaks. Hence, elective laparoscopic colonic surgery should be favored due to fewer post-operative complications and superior short-term outcomes. Larger studies on colon cancer are imperative for enhanced healthcare delivery.

## INTRODUCTION

Colorectal cancer (CRC), also known as colon cancer or large bowel cancer, encompasses cancerous growths in the colon, rectum, and appendix.^1^-^3^ It is a significant global health concern, ranking as the third most common malignancy and the second leading cause of cancer-related deaths. In 2020, the CRC was responsible for an estimated 1.9 million new cases and 0.9 million deaths worldwide.^4^ Colon cancer is a prevalent malignancy in Pakistan, ranking as the fourth most common cancer in the country. Its incidence not only leads to a substantial burden of morbidity but also contributes to increased mortality rates.^5^ It has an incidence ranging from 4% to 6.8% across different regions.^6^ Risk factors associated with an increased incidence of CRC include the consumption of animal products, smoking, binge drinking, and inflammatory bowel disease. Additionally, rising obesity rates and decreased physical activity levels contribute to this risk.^7^-^9^

The survival rate of colon cancer varies based on factors such as TNM staging and early detection, ranging from 90% to 14%.^10^ Despite advancements in neoadjuvant and adjuvant treatments, curative surgical excision remains the primary therapeutic approach.^11^ Among surgical modalities, laparoscopic procedures have become the preferred choice for treating these cancers. The survival rate is influenced by TNM staging, early detection, and prognostic factors such as age and tumour differentiation.^12^

However, establishing laparoscopic treatment of colonic tumours as the standard of care worldwide presents challenges. Countries like Pakistan face financial constraints and limited opportunities for surgeons to acquire the necessary education and training.^10^ Therefore, this study aimed to investigate elective colon cancer resections performed at a newly developed tertiary care cancer centre, focusing on demographic characteristics and short-term outcomes.

## METHODS

In April 2021, surgical services were initiated at the Shaukat Khanum Memorial Cancer Institute and Research Centre in Peshawar. After obtaining consent from patients and ethical approval from the ethics committee (Ref. EX-04-06-22-01-AI Date February 03 2023), we conducted a retrospective evaluation of data pertaining to elective laparoscopic colorectal resections performed between April 2021 and February 2022. The study included a total of 157 cases, out of which 79 were colon cancer cases. Only patients who were 18 years of age or older and had confirmed colon cancer through biopsy were included, while others were excluded.

### Statistical Analysis:

The collected data were subjected to analysis using SPSS-22. Descriptive statistics and cross-tabulations were utilized to assess short-term outcomes, and the results were presented in the form of tables and charts.

## RESULTS

The study focused on patients with colon cancer and included a total of 79 participants. The demographic distribution of the sample is presented in Table-I. The majority of patients, 26 (33%), fell within the age group of 36-45 years. This was followed by 24 patients (30.7%) aged between 46 and 55 years, 14 patients (13.9%) in the 56-65 age group, and 13 patients (16.4%) in the 25–35 age group. In terms of gender, 61 (77.2%) participants were males, while 18 (22.7%) were females. Additionally, 55 (69.6%) patients were from the Khyber Pakhtunkhwa (KPK) region, 19 (24%) were from Afghanistan, 3 (3.7%) were from the Federally Administered Tribal Areas (Fata), and 1 (1.2%) was from Punjab. Table-I. This also shows the distribution of ASA scores among patients who underwent laparoscopic colon cancer surgeries at a newly developed cancer centre in Peshawar, Pakistan. The majority of patients (86%) had an ASA score of 2, while 11.3% had an ASA score of 3. One patient each had an ASA score of one and four. Table-I.

**Table-I:** Baseline Patient Demographics.

Variable	Value (n = 79)
***Age Group (n, %)***	
25-35	13 (16.4)
36-45	26 (33)
46-55	24 (30.3)
56-65	14 (17.7)
66 and above	2 (2.5)
***Gender (n, %)***	
Male	61 (77.2)
Female	18 (22.8)
***Regional distribution***	
KPK	55 (71)
Afghanistan	19 (24)
Fata	3 (3.8)
Punjab	1 (1.2)
***ASA Classification (n, %)***	
ASA I	1 (1.2)
ASA II	68 (86)
ASA III	9 (11.4)
ASA IV	1 (1.2)
***Location of the tumor (n, %)***	
Ascending colon:	24 (30.4)
Descending colon:	6 (7.6)
Transverse colon:	6 (7.6)
Splenic flexure:	7 (8.8)
Hepatic flexure:	21 (26.6)
Sigmoid colon	15 (19)
***Number of lymph nodes retrieved (n, %)***	
Less than 12	3 (3.8)
More than 12	76 (96.2)
***Number of positive lymph nodes (n, %)***	
0-5	68 (86)
6-10	8 (10.1)
11-20	1 (1.4)
21 and above	2 (2.5)
***Histopathological Grading (n, %)***	
Poorly differentiated	19 (24)
Moderately differentiated	56 (71)
Well-differentiated	4 (5)

Among the patients, the most common cancer site was the ascending colon 24 (30.3%), followed by the hepatic flexure 21 (26.5%), sigmoid colon 15 (18.9%), splenic flexure 7 (8.8%), and both the descending and transverse colon 6 (7.5% each). The mean number of retrieved lymph nodes was 19.0, with most patients having 0-5 positive lymph nodes (68 cases), followed by 6-10 positive lymph nodes (eight cases). The most frequently performed procedures were right hemicolectomy (39.2%), extended right hemicolectomy (25.3%), and sigmoid colectomy (18.9%). Histopathological Grading (Table-I) revealed that out of the total cases, 56 were moderately differentiated, 19 were poorly differentiated, and four were well-differentiated tumors. Regarding the resection status, 77 patients (97.4%) achieved R0 resection, indicating complete removal of the tumor, while two patients (2.6%) had R1 resection. Pathological TNM staging revealed that the majority of the tumors observed were classified as T3, followed by T4 and T2. (Table-II)

**Table-II:** Shows Clinical versus pathological TNM.

	T					N			M		
	0	1	2	3	4	0	1	2	0	1	X
Clinical:	2		1	59	4a: 15 4b: 2	7	42	29 2b: 1	76	1a: 2	1
Pathological:	3		5	61	4a: 7 4b: 3	40	1a: 13 1b: 7 1c: 2	2a:8 2b: 9	67	1a: 2 1c: 2	8

The outcomes of laparoscopic colon cancer surgeries, comparing patients with colon cancer (n = 79) and those with other diagnoses (n = 78) other diagnoses included benign conditions like diverticulosis, solitary rectal ulcers and angiodysplasias. Table-III. The study examined various short-term outcome measures and their statistical significance. Operating time showed a non-significant difference between the two groups, that is between the colorectal cancer patients having a mean duration of 249 minutes (SD = 13.4-29.1) and patients with other diagnoses (benign conditions) having 231 minutes (SD = 9.71-17.32). Similarly, there were no significant differences in postoperative ICU care or conversion to open laparotomy. However, colon cancer patients had a higher incidence of intraoperative bleeding compared to patients with other diagnoses, with a significant association observed. The need for blood transfusion, re-look laparoscopy, 90-day mortality, death within 30 days after surgery, and disease-free status did not show significant differences between the two groups. Notably, loco-regional tumors and distant metastases had a significant relationship, with colon cancer patients exhibiting higher rates.

**Table-III:** Short-Term Outcomes of Laparoscopic Colon Cancer Surgeries.

Outcomes	Colon Cancer (n = 79)	Rectal Cancer (n = 78)	P- value
Operating Time (mean ± SD)	249 ± 13.4-29.1	231 ± 9.71- 17.32	0.091
Postoperative ICU Care (n, %)	10 (12.6%)	7 (8.9%)	0.472
Conversion to Open Laparotomy (n, %)	1 (1.2%)	3 (3.8%)	0.921
Intraoperative Bleeding (n, %)	1 (1.2%)	2 (2.5%)	0.036
Blood Transfusion Required (n, %)	9 (11.3%)	7 (8.9%)	1.012
Re-look Laparoscopy (n, %)	1 (1.2%)	0 (0%)	1.301
90-day mortality (n, %)	2 (2.5%)	3 (3.8%)	0.040
Death 30 days after surgery (n, %)	2 (2.5%)	1 (1.2%)	0.006
Disease-free (n, %)	53 (67%)	62 (79.4%)	0.716
Loco-regional tumor and distant mets (n, %)	26 (33%)	21 (26.9%)	0.023

## DISCUSSION

Laparoscopic surgery has shown promising results in terms of reduced morbidity and mortality and improved completeness of cancer resection in previous research. A population-based study conducted in Sweden compared laparoscopic (LAP) and open (OPEN) surgeries for colon cancer, revealing the advantages of LAP in terms of short-term mortality, morbidity, and completeness of cancer resection.^13^ This finding is consistent with our present study, which focused on laparoscopic procedures and reported low mortality rates and favorable short-term outcomes. Furthermore, laparoscopic surgery for colon cancer has been extensively researched and proven to be oncologically safe, offering various benefits such as reduced blood loss, less pain, shorter hospital stays, and improved quality of life compared to open surgery.^14^-^16^

A retrospective case-control study comparing laparoscopic-assisted and open surgery for colon cancer reported similar short-term outcomes but found that laparoscopic surgery had a shorter hospital stay, less intraoperative blood loss, and a faster return of bowel function.^17^ While our study did not directly compare laparoscopic and open surgeries, we did report the mean operating time and highlight the need for postoperative ICU care, conversions to open laparotomy, and intraoperative bleeding complications.

Studies evaluating the outcomes of laparoscopic surgery in elderly patients with colon cancer have demonstrated its safety and curative potential. A retrospective cohort study comparing laparoscopic surgery in elderly patients (≥80 years old) with non-elderly patients found similar short- and long-term outcomes, suggesting that laparoscopic surgery is safe and effective in elderly patients.^18^ Few other studies have also showed similarity with the results of this study, promoting the use of the laparoscopic approach for colon cancer surgeries has shown better short come outcomes irrespective of age.^19^-^20^ Although our study did not specifically focus on elderly patients, the reported short-term outcomes provide valuable information in accordance with the mentioned study in a broader context.

Our study’s findings are similar to previous research on laparoscopic colon cancer surgeries. Laparoscopic surgery demonstrates advantages in terms of short-term outcomes, including reduced morbidity and favorable completeness of cancer resection. These results contribute to the existing body of evidence supporting the oncological safety and benefits of laparoscopic surgery. Nevertheless, further studies comparing laparoscopic and open surgeries, as well as investigating long-term outcomes, are necessary to develop a comprehensive understanding of laparoscopic colon cancer surgeries.

## CONCLUSION

Our study confirms the benefits of laparoscopic colon cancer surgeries, showing reduced morbidity and improved cancer removal. It highlights the safety and advantages, such as less blood loss, lower pain, and shorter hospital stays. While more research comparing different surgeries is needed, our findings inform clinical decisions and enhance patient outcomes in managing colon cancer.

### Authors’ Contribution:

**MFS, RA, HJA:** Concept and design. **IIN, RA, MFS, HJA:** Acquisition, analysis and interpretation of data. **MFS, IIN, HJA, RA:** Critical review of the manuscript for important intellectual content. **HJA, RA, IIN:** Drafting of the manuscript. **RA:** Supervision. All authors have reviewed the final version to be published and agreed to be accountable for all aspects of the work.
